# Probiotics in *Naunyn–Schmiedeberg’s Archives of Pharmacology*

**DOI:** 10.1007/s00210-021-02110-5

**Published:** 2021-06-25

**Authors:** Detlef Neumann, Roland Seifert

**Affiliations:** Institute of Pharmacology, Hannover Medical School, Carl-Neuberg-Str. 1, 30625 Hannover, Germany

Probiotics, which are used in the management of health and disease, are currently an emerging topic in the scientific literature in general as well as in pharmacological journals (Fig. 1). Therefore, the question arises whether the scientific evaluation of probiotics is a topic relevant for pharmacology. More specifically, we have to ask the question whether *Naunyn–Schmiedeberg’s Archives of Pharmacology* (NSAP) is a proper journal to publish articles on probiotics. This question has been recently discussed within the Editorial Board since we already received submissions of this topic. Several submissions have been rejected, but one paper has been recently published (Patel et al. 2020). The rejections were, in part, due to the fact that criteria for submissions on probiotics to this journal have not yet been officially defined.

**Fig. 1:**
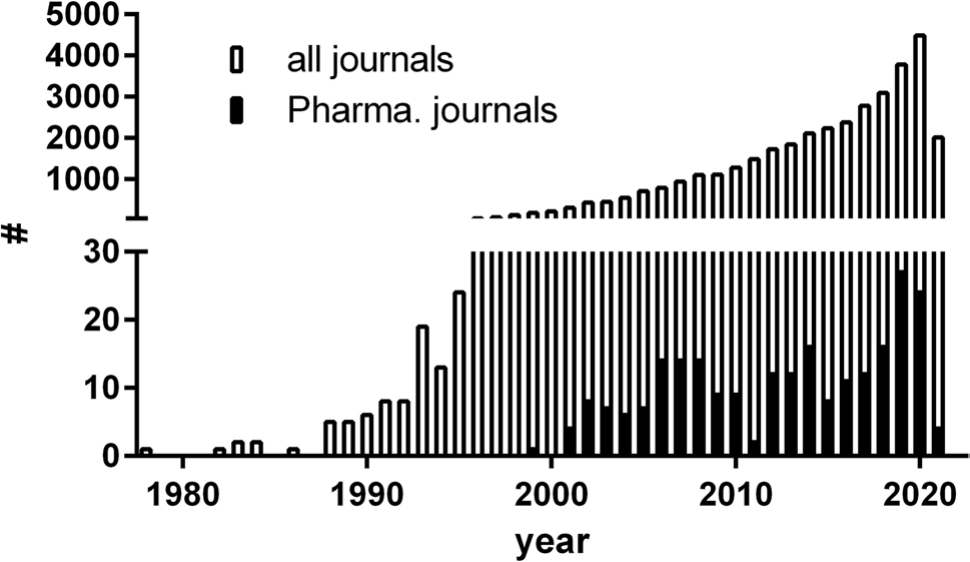
Number of publications on probiotics in PubMed.org. The search strings “probiotic” (all journals) or ‘((“European journal of pharmacology”[Journal]) OR (“The Journal of pharmacology and experimental therapeutics”[Journal]) OR (“Biochemical pharmacology”[Journal]) OR (“British journal of pharmacology”[Journal]) OR (“Journal of pharmaceutical sciences”[Journal]) OR (“Naunyn–Schmiedeberg’s archives of pharmacology”[Journal]) OR (“Basic clinical pharmacology toxicology”[Journal]) OR (“Clinical pharmacology and therapeutics”[Journal]) OR (“Experimental and therapeutic medicine”[Journal]) OR (“Alimentary pharmacology therapeutics”[Journal])) AND (probiotic)’ (Pharma. Journals) was used to search PubMed.org on June 2, 2021

Therefore, we would like to provide official editorial guidelines to our authors on criteria for papers on probiotics to be considered for publication in NSAP. To the best of our knowledge, this is one of the first editorials in pharmacological journals defining the criteria required for submissions of studies on probiotics.

Pharmacology is the scientific discipline analyzing the effects of a pharmacon (more commonly referred to as drug) on a living organism, and vice versa (Seifert 2019). A pharmacon is any artificial or natural substance that affects a living organism’s physiology at the cellular, tissue, or organ level. Thus, pharmacology is the science studying the interactions between a biologically active substance and a living organism. To discriminate between pharmacology and toxicology, it may be added that the pharmacon supports the organism’s health status and does not harm it. This is, however, a very weak discriminatory line since it is not only a matter of the pharmacon’s nature, but also of its concentration and mode of application (Seifert 2019). Regarding the initial question, we consequently have to ask whether probiotics are a substance that interacts with a living organism.

According to the Food and Agriculture Organization/World Health Organization (FAO/WHO) definition, probiotics are live (micro)organisms (mostly bacteria and yeasts) that, when administered in adequate amounts, confer a health benefit on the host (Hill et al. 2014). Probiotics confer a health benefit to the living organism (the host) by affecting its physiology. Also, the host affects the probiotic’s physiology, at least by final excretion (permanent colonization is very rare), rendering it a true interaction as required by the definition of pharmacology.

Thus, eventually the question remains whether the term “substance” includes live (micro)organisms. Commonly, substance is meant to be a chemically defined material excluding probiotics from the definition. However, we may consider probiotics not just as pharmacologically active substance, but rather a specific formulation of microorganisms, constituting a medicine or medicament. Thus, probiotics contain the active substance formulated in a series of supportive compounds. Indeed, although we just start to understand the effects of probiotics by defining the specific microbial species that provide a therapeutic benefit in a pathological situation, it is most probable that defined microbial molecular structures, but not the entire probiotic microorganism, confer the beneficial effect (Fig. 2). In essence, probiotics are mixtures of substances forming a medicine or medicament. Thus, the task of pharmacology would be the identification of the “substance” transported by the probiotics, and its scientific evaluation, very similar to the field of “herbal medicine” (Merfort et al. 2017). In conclusion, the integration of scientific articles handling probiotics in NSAP is, in principle, justified.

**Fig. 2:**
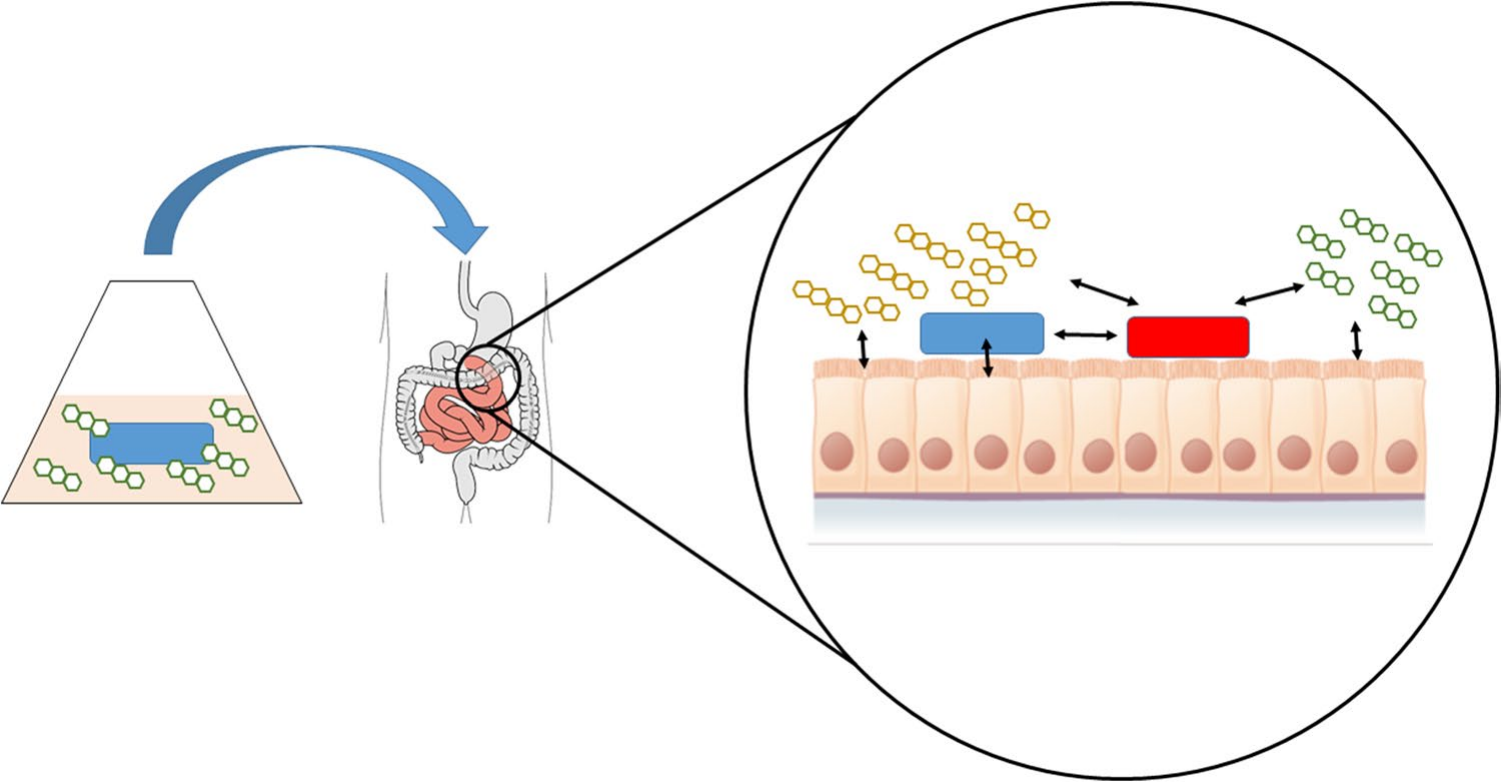
Proposed mechanism of probiotics effects on the host. Probiotics (blue) are fermented for multiplication and activation. Thereby, they produce possibly active substances (green 3-cyclic symbols = “postbiotica”). After ingestion, both can interact with the host’s epithelia and/or endogenous microbiota (red). Interaction of the microbiota with the host’s epithelia or microbiota can be performed either by direct cell–cell contacts via cell surface molecules or by substances produced specifically in the host’s gastrointestinal microenvironment (yellow 2- and 4-cyclic symbols)

Papers dealing with probiotics to be considered for publication in NSAP must fulfill ten essential criteria:

1. In analogy to a chemically defined drug, i.e., a substance, authors using probiotics have to exactly define the microbial strains present in their preparations and
2. to quantify the applied amount.
3. Moreover, the route of application,
4. the number of applications,
5. and its intervals,
6. as well as the incubation period have to be specified.
7. Clearly defined parameters of pharmacodynamics have to be evaluated as readouts.
8. Lastly, if possible, solely descriptive studies should be avoided,
9. at least the evaluation of some mechanistic insight regarding the probiotics-induced observation is expected.
10. Authors must clearly outline unknown facets of the effects of probiotics and suggest appropriate future studies to address these issues.

In an exemplary paper published in 2020 in NSAP (Patel et al. 2020), the above criteria have been sufficiently fulfilled.

In conclusion, *Naunyn–Schmiedeberg’s Archives of Pharmacology* welcomes papers in the emerging field of probiotics (Fig. 1) that follow the above-listed ten scientific criteria and particularly aim at elucidating the complex mechanisms underlying the pharmacological effects of probiotics (Fig. 2).

## References

[CR1] Hill C, Guarner F, Reid G (2014). Expert consensus document. The International Scientific Association for Probiotics and Prebiotics consensus statement on the scope and appropriate use of the term probiotic. Nat Rev Gastroenterol Hepatol.

[CR2] Merfort I, Michel MC, Seifert R (2017). Revised editorial guidelines for manuscripts on the pharmacology of plant extracts. Naunyn Schmiedebergs Arch Pharmacol.

[CR3] Patel C, Pande S, Acharya S (2020). Potentiation of anti-Alzheimer activity of curcumin by probiotic Lactobacillus rhamnosus UBLR-58 against scopolamine-induced memory impairment in mice. Naunyn Schmiedebergs Arch Pharmacol.

[CR4] Seifert R (2019). Basic knowledge of pharmacology.

